# Polysaccharides from aloe vera target the Wnt/β-catenin pathway to impact the tooth density of pulpitis rats

**DOI:** 10.1590/acb371202

**Published:** 2023-01-13

**Authors:** Ling Jiang, Yang Lu, Hongyan Zhao, Weiyang He

**Affiliations:** 1 MD. Chongqing Medical University – Department of Urology Surgery – The First Affiliated Hospital – Chongqing, China.; 2 MD. Chongqing University – Department of Plastic Surgery – Central Hospital – Chongqing, China.

**Keywords:** Polysaccharides, Aloe, Pulpitis, Osteoblasts, Rats

## Abstract

**Purpose:**

To investigate the mechanism of polysaccharides from aloe vera (PAV), a main active ingredient of *Aloe vera*, treatment in pulpitis rats.

**Methods:**

Pulpitis were modeled by drilling the occlusal central fossa with Sprague Dawley rats. Next, the rats were treated with 20, 40, and 80 mg/kg PAV for three weeks, respectively. Computed tomography scanning assay, hematoxylin and eosin staining, and tartrate-resistant acid phosphatase staining were used to detect the pathology change. Then, levels of tumor necrosis factor-α, interleukin-1β, prostaglandin E_2_, and ciclooxigenase 2 were detected by enzyme-linked immunosorbent assay. The expressions of bone morphogenetic protein 2 human (BMP-2), osteocalcin, osterix, and runt-related transcription factor 2 (Runx2) were quantified by quantitative real-time polymerase chain reaction and Western blotting (WB). Finally, Wnt3a expression, p-GSK3β/GSK3β and p-β-catenin/β-catenin ratio were analyzed by WB.

**Results:**

PAV up regulated the bone mineral density, and reduced the breakage of the crown and cervical structures, and the necrosis of the crown and root pulp of pulpitis rats. In addition, results indicated that PAV could inhibit osteoblast formation. While osteoblasts’ number was decreased, proteins of BMP-2, osteocalcin, osterix, and Runx2 were up-regulated by PAV. Furthermore, PAV increased the Wnt3a expression and the p-β-catenin/β-catenin ratio, and decreased p-GSK3β/GSK3β ratio. Interestingly, these effects were all in dose dependence.

**Conclusions:**

PAV could inhibit pulp inflammation and promote osteoblasts differentiation via suppressing the activation of the Wnt/β-catenin signaling, enhancing the dental bone density.

## Introduction

The pulp is the innermost part of the tooth structure and originates from the nerve spines, which are composed of connective tissue, neuronal cells, endothelial cells, and undifferentiated mesenchymal stem cells^1^. Pulpitis is a common oral disease that usually yields intolerable pain when in an acute outbreak. In addition, tooth inflammation causes pulp destruction and finally leads pulp function loss^2^. At present, root canal therapy is one of the most important treatments for pulpitis^3^^,^ 4. However, the attendant drawbacks, such as brittle dentition, have led to the exploration of new areas of treatment methods^5^^,^ 6. Thus, increasing tooth bone density and suppressing pulp inflammation are the treatment guidelines for pulpitis.

In recent years, osteoblast’s role has attracted increasing attention in pulpitis^7^. The Wnt/β-catenin signaling pathway plays an important role in osteogenesis, while it regulates osteoblast differentiation^8^. In order to prevent tooth brittle and bone loss caused by pulpitis, it is necessary to inhibit the formation of osteoclasts. Nevertheless, persistent pulpal inflammation hinders this process^9^. As reported, DKK1, a kind of inhibitor of the Wnt signaling pathway, is up regulated by inflammatory cytokines, and binds with the Wnt receptor of osteogenic precursor cells. Finally, blocking the Wnt signaling pathway inhibits osteoblast differentiation^10^. It follows that the Wnt signaling pathway and inflammatory cytokines together influence the progression of pulpitis.

*Aloe vera* is a perennial herb of Liliaceae. The main active ingredient, polysaccharides from aloe vera (PAV), is a class of macromolecular compounds, which is mainly composed of mannose, galactose, glucose, xylose, arabinose, and rhamnose^11^. As reported, PAV has a variety of pharmacological effects, including cognitive improvement, immunity improvement, anti-tumor, wound repair, and anti-viral effects^12^^,^ 13. Considering the pharmacologic action of PAV, it is necessary to study the PAV effects on pulpitis treatment, since osteoclast and inflammatory cytokines are essential for reparative pulpitis. This study aimed to base on the Wnt/β-catenin signaling pathway to investigate the effects of osteoblast differentiation and inflammation on pulpitis rats via PAV treatment.

## Methods

### Animal model establishment and drug treatment

A total of 30 female Sprague Dawley rats was used in this experiment. After a week of adaptive rearing, modeling pulpitis rats as follow. First, rats were anesthetized with 0.1% ketamine hydrochloride injection (Heng Rui Pharmaceutical Co. LTD., China) by intraperitoneal injection. Considered no limb reaction and no conjunctival reflex as successful anesthesia. The oral was alternatingly douched with 3% hydrogen peroxide and physiological saline, and disinfected with an iodophor. Then, the occlusal central fossa was drilled by a high-speed turbine (Sirona Co. LTD., Germany) and FG1/4 ball drill to explore pulp under water cooling. Finally, the DG16 probe penetrated the roof of the pulp chamber until appearing bleeding point. On the second day after modeling, 20, 40, and 80 mg/kg doses of PAV were used to feed pulpitis rats for three weeks. PAV was obtained in Shanghai Macklin Biochemical Co., Ltd (lot#:C10433991).

### Computer tomography scanning assay

All rats were executed, and maxillary bone tissue was separated. SCANCO Medical μCT 50 (SCANCO Medical AG Co. LTD., Swiss) was used to scan maxillary bone tissue. The scanning parameters were as follow: 70 kV, 200 uA, 300 ms, 15 um, and 0.5 m AI.

### Hematoxylin and eosin and TRAP staining assay

The drilled tooth was fixed with 10% neutral formaldehyde. Then, samples were decalcified by 15% EDTA. After that, they were dehydrated in a graded ethanol/xylene series and washed with phosphate buffer saline. Next, the samples were embedded in paraffin and cut into 5-μm thick slices. Up to now, samples were prepared to stain. Hematoxylin and eosin (HE) were used for HE staining, and a tartrate-resistant acid phosphatase (TRAP) staining kit (Sigma, St. Louis, MO, USA) was used for TRAP staining, which was performed by the manufacturer’s protocol. Finally, they were examined under a light microscope.

### Real-time fluorescence quantitative polymerase chain reaction assay

The drilled tooth was used to extract total RNA by TRIzol reagent (Invitrogen, USA). mRNA of bone morphogenetic protein 2 human (BMP-2), osteocalcin, osterix, and runt-related transcription factor 2 (Runx2) were quantified. Samples were conducted by the SYBR Green assay (Vazyme, China) and 2-ΔΔCt method, which was based on the manufacturer’s protocol. Primers were shown as follows:

- BMP-2: forward primer: 5’-CCA AAC ACA AAC AGC GGA AGC GTC TT-3’, and reverse primer: 5’-GGC ATG GTT GGT GGA GTT CAG GTG AT-3’;
- Osteocalcin: forward primer: 5’-GAC TGC ATT CTG CCT CTC TG-3’, and reverse primer: 5’-ATT CAC CAC CTT ACT GCC CT-3’;
- Osterix: forward primer: 5’-CAC CCA TTG CCA GTA ATC TTC GT-3’, and reverse primer: 5’-GGA CTG GAG CCA TAG TGA GCT TCT-3’;
- Runx2: forward primer: 5’- ATC CAT TCC ACC ACG CCG CTG TCT-3’, and reverse primer: AGC ACC TGC CTG GCT CTT CTT ACT GA-3’;
- β-actin: forward primer: 5’-ACC GTG AAA AGA TGA CCC AGA T-3’, and reverse primer: 5’-AGC CTG GAT GGC TAC GTA CAT G-3’;
- β-actin acted as a control.

The real-time quantitative polymerase chain reaction (RT-qPCR) was performed at 95 °C, 10 min; 35 cycles for 95 °C-2 s, and 60 °C-30 s. The melting curve stage was performed at 95 °C-30 s, 60 °C-30 min, and 95 °C-30 s.

### Enzyme-linked immunosorbent assay

The levels of tumor necrosis factor-α (TNF-α), interleukin-1β (IL-1β), prostaglandin E_2_ (PGE2), and ciclooxigenase 2 (COX-2) were quantified in the serum of rats by enzyme-linked immunosorbent assay (ELISA) kits. ELISA kits were as follows: IL-1β ELISA kits (ZC-37974, ZCi Bio, China); TNF-α ELISA kits (ZC-35733, ZCi Bio, China); Rat PGE2 ELISA Kit (ZC-37100, ZCi Bio, China); and Rat COX-2 ELISA kit (ZC-36713, ZCi Bio, China). Experimental procedures followed the manufacturer’s instructions. All experiments were performed in triplicate.

### Western blotting assay

The drilled tooth was prepared with RIPA cell lysis buffer, the supernatants were collected, and the total protein by the BCA kit (Thermo Scientific, Rockford, IL, United States of America) was measured. It was added 20 μg total protein per line to separate by sodium dodecyl sulfate-polyacrylamide gel (SDS-PAGE). Then, proteins were transferred onto polyvinylidene difluoride (PVDF) membranes. PVDF membranes were blocked in TBS-T at room temperature for 2 h and washed three times. Next, they were incubated with primary antibodies at 4 °C overnight.

Primary antibodies included that BMP-2 (1:1,000, ab225898, Abcam), osteocalcin (1:2,000, ab76956, Abcam), osterix (1:1,000, ab209484, Abcam), and Runx2 (1:500, ab76956, Abcam), Wnt3a (1:1,000, ab219412, Abcam), p-GSK3β (1:1,000, #5558, CST), GSK3β (1:1,000, #12456, CST), p-β-catenin (1:1,000, #2009, CST), β-catenin (1:1,000, #8480, CST) and GAPDH (1:5,000; #5174, CST). Then, membranes were incubated with goat anti-mouse IgG (H+L) HRP (1:5,000, ab6789, Abcam) and goat anti-rabbit IgG (H+L) HRP (1:5,000, s0001, Affinity) secondary antibodies for 2 h at room temperature and washed three times. PVDF membranes were detected with ECL, and the protein bands were quantified by Scion Image 4.0 software (Scion Corporation, Frederick, MD, United States of America).

### Statistical analysis

GraphPad Prism 9.1.2 (GraphPad Software, Inc.) was operated to analyze the data and generate charts. All data are expressed as the mean ± standard deviation and all tests in triplicate. The two groups were analyzed by T-test, unpaired. One-way analysis of variance (ANOVA) was used to analyze groups among three. It was considered statistically significant when P < 0.05.

## Results

### PAV enhanced the bone mineral density of pulpitis rats

**Figure 1:**
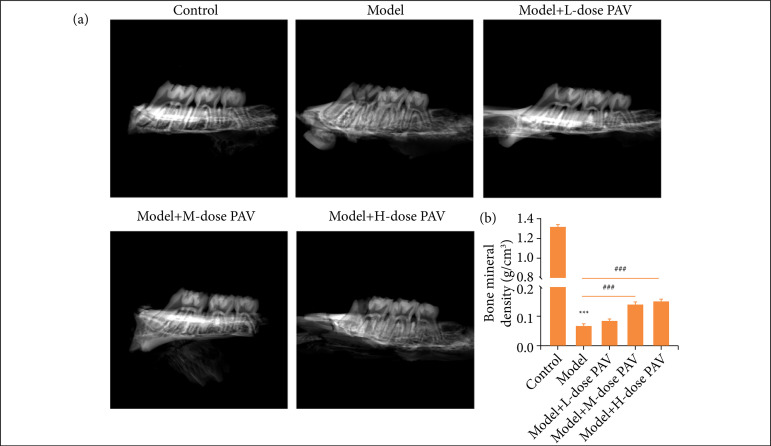
Polysaccharides from aloe vera (PAV) enhanced the bone mineral density of pulpitis rats. Respectively, 20 mg/kg **(L)**, 40 mg/kg **(M)** and 80 mg/kg **(H)** PAV were used to treat pulpitis rats for three weeks. **(a)** Computer tomographyimage of dental pulp for rats. **(b)** Bone mineral density was quantified. Bars represent the mean ± standard deviation.

The occlusal central fossa was drilled to explore pulp and treated with the different doses of PAV. Finally, the maxillary bone of rats was scanned by computer tomography. As Fig. 1a shows, the three teeth had a similarly grey level. However, the model group displayed that the gray level of the first medullated tooth on the left was significantly lower than that other two. With the PAV dose increasing, the gray level of the three teeth on the maxilla gradually showed consistency. The bone mineral density showed the same trend (Fig. 1a). Results explored that pulp exposure could induce severe bone density loss, and high-dose PAV could relieve it.

***p < 0.001, compared with the control group; ###p < 0.001, compared with the model group.

### The effects of PAV on the pathology of pulpitis in rats

**Figure 2:**
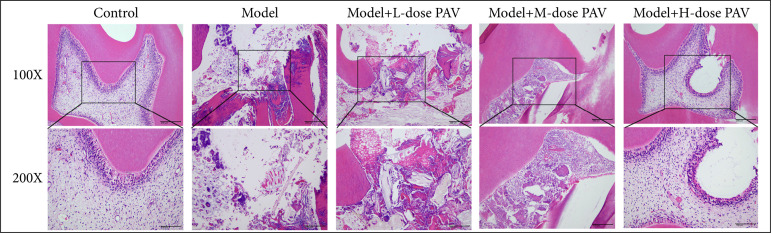
The effects of polysaccharides from aloe vera (PAV) on the pathology of pulpitis in rats. Respectively, 20, 40, and 80 mg/kg PAV were used to treat pulpitis rats for 3 weeks. The dental pulp was stained for hematoxylin and eosin staining.

To explore the effects of PAV on the pathology change of pulpitis in rats, the dental pulp was stained for HE staining. As Fig. 2 shows, in the control group, no inflammatory cell infiltration was seen in the field, the adult dentin cells were arranged regularly, and the pulp chamber was with no abnormal pulp cells. Compared with the control group, the model group mainly manifested pathology as follows: breakage of the crown and cervical structures, necrosis of the crown pulp, and root pulp near the root canal opening. After PAV treatment, with the PAV dose increasing, the breakage of the crown and cervical structures was decreasing, and the necrosis of the crown and root pulp was decreasing too. Particularly, the high-dose PAV showed the best treatment effects.

Bars = 200 μm (100×) and bars = 100 μm (200×).

### The effects of PAV on osteogenesis in pulpitis rats

**Figure 3:**
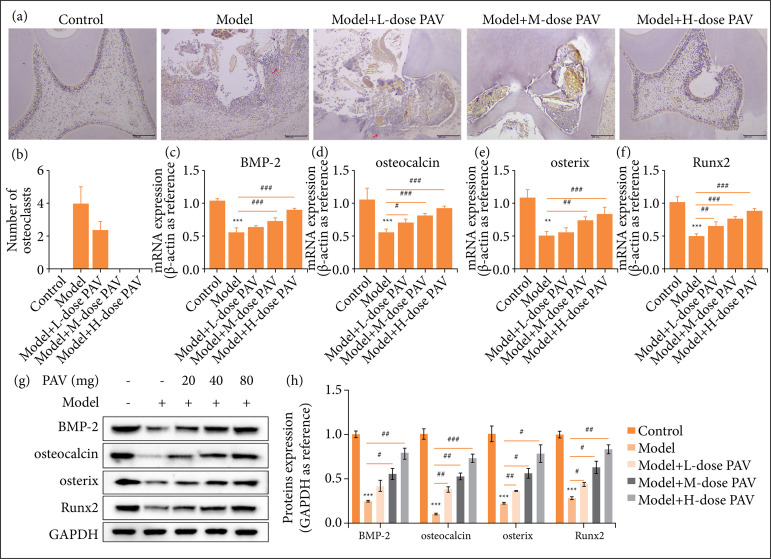
The effects of polysaccharides from aloe vera (PAV) on the osteogenesis and the expression of osteogenesis-related genes in pulpitis rats. Respectively, 20, 40, and 80 mg/kg PAV were used to treat pulpitis rats for three weeks. **(a)** TRAPstaining was performed to detect osteoclast, bars = 200 μm (100×). **(b)** The number of osteoclasts was counted perfield of the microscope. The osteogenesis-related genes including **(c)** bone morphogenetic protein 2 human(BMP-2), **(d)** osteocalcin, **(e)** osterix, and **(f)** Runx2 were quantified by quantitative real-time polymerasechain reaction. Western blotting was performed to quantify the osteoclast-related proteins **(g)** andanalyzed by bar chart **(h)** Bars represent the mean ± standard deviation.

The effects of PAV on osteogenesis were measured by TRAP staining, qRT-PCR, and Western blotting (WB) in pulpitis rats. Osteoblasts (red arrow) as shown as purple-red (Fig. 3a), and they counted as per the field of the microscope in Fig. 3b. The osteoblasts number between the Model group and the Model+L-dose PAV group had no significant difference when other groups were zero. qRT-PCR was performed to quantify the mRNA levels of osteogenesis-related genes. Expression of BMP-2 (Fig. 3c), osteocalcin (Fig. 3d), osterix (Fig. 3e), and Runx2 (Fig. 3f) showed a similar trend. For instance, compared with the control group, the BMP-2 expression was down-regulated, and PAV reversed it with dose dependence (Fig. 3c). Meanwhile, we used the WB to verify the proteins of the above genes (Fig. 3f). Interestingly, the protein expressions agreed with the results of qRT-PCR (Fig. 3g).

**p < 0.01; ***p < 0.001, compared with the control group; #p < 0.05; ##p < 0.01; ###p < 0.01, compared with the model group.

### Pav inhibited the inflammation of pulpitis rats

**Figure 4:**
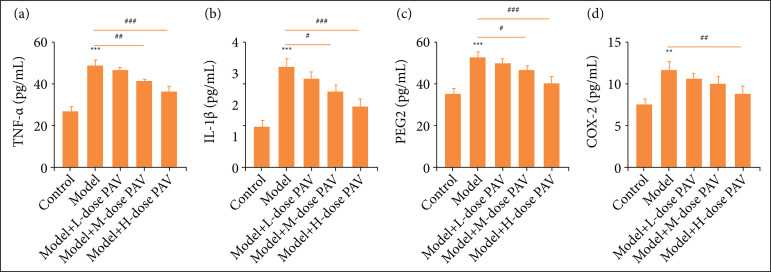
Polysaccharides from aloe vera (PAV) inhibited the inflammation of pulpitis rats. Respectively, 20, 40, and 80 mg/kg PAV were used to treat pulpitis rats for three weeks. Enzyme-linked immunosorbent assay was performedto quantify the expression of **(a)** tumor necrosis factor-α (TNF-α), **(b)** interleukin-1β (IL-1β), **(c)** prostaglandinE_2_ (PGE2), and **(d)** ciclooxigenase 2 (COX-2). Bars represent the mean ± standard deviation.

It is well known that inflammation largely affects the formation of osteoblasts. We performed ELISA to detect the inflammation of pulpitis rats under PAV treatment, which explored PAV function. As Figs. 4 a-4d show, compared with the control group, the model group showed significantly higher TNF-α, IL-1β, PGE2, and COX-2 levels. However, 40 and 80 mg/kg PAV treatment caused reduction of TNF-α, IL-1β, and PGE2 levels with a significant difference. While 20 mg/kg PAV treatment had no significant effects on these inflammatory factors, 40 mg/kg PAV treatment could not affect COX-2 levels either.

**p < 0.01; ***p<0.001, compared with the control group; #p < 0.05; ##p < 0.001; ###p < 0.01, compared with the model group.

### The effects of PAV on the Wnt/β-catenin pathway in pulpitis rats

**Figure 5:**
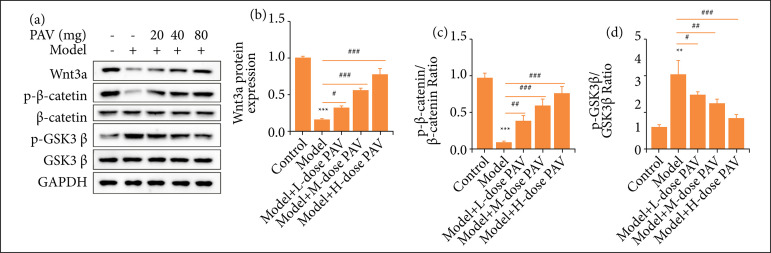
The effects of polysaccharides from aloe vera (PAV) on the Wnt/β-catenin pathway in pulpitis rats. Respectively, 20, 40, and 80 mg/kg PAV were used to treat pulpitis rats for three weeks. **(a)** Western blotting was performed to quantifythe Wnt/β-catenin pathway-related proteins. **(b)** Wnt3a protein expression, **(c)** p-β-catenin/β-catenin ratio,**(d)** p-GSK3β/GSK3β ratio were analyzed by the bar chart. Bars represent the mean ± standard deviation.

Wnt/β-catenin pathway-related proteins were measured by WB, proteins including Wnt3a, p-GSK3β, GSK3β, p-β-catenin, and β-catenin, while GADPH was the reference (Fig. 5a). Fig. 5b shows that the expression of Wnt3a was down-regulated significantly after pulpitis modeling, but PAV treatment reversed the situation with dose dependence. In addition, thepβ--catenin/β-catenin ratio exhibited a similar trend to Wnt3a (Fig. 5c). On the contrary, compared with the control group, the p-GSK3β/GSK3β ratio of the model group showed a significantly higher level, and it decreased after PAV treatment. Furthermore, 80 mg/kg PAV had the most down regulation (Fig. 5d).

**p < 0.01; ***p < 0.001, compared with the control group; #p < 0.05; ##p < 0.001; ###p < 0.01, compared with the model group.

## Discussion

Previously, Budai *et al*.^14^ investigated that *Aloe vera* could reduce the inflammatory cytokine and NLRP3 inflammasome production induced by lipopolysaccharide (LPS). Meanwhile, the inflammation classical pathway, the NF-κB pathway is also blocked by it^14^. Cowan^15^ summarized that *Aloe vera* could be an anti-inflammatory agent for osteoarthritis treatment to alleviate pain^5^. *Aloe vera* was also reported having multiple pharmacological effects, including anti-diabetic^16^, anti-tumor^17^, and anti-oxidant^18^. As the main active ingredient of *Aloe vera*, researchers have suggested PAV has similar effects^19^^,^ 20. The present study indicates that PAV can inhibit pulp necrosis and enhance the bone density of tooth crowns. Moreover, PAV showed anti-inflammation and inhibition of osteoclast formation in pulpitis mice. Results are consistent with previous reports. It suggested that PAV may act as a potential drug for pulpitis treatment.

COX is a key enzyme for the synthesis of prostaglandins (PGs), with two isoforms: COX-1 and COX-2. Particularly, COX-2 catalyzes the PGE2 formation in response to proinflammatory cytokines (IL-1β and TNF-α)^21^. The PGE2 is associated with pain and caused inflammation enhancement^22^. Therefore, the expressions of TNF-α, IL-1β, PGE2, and COX-2 reflected inflammation status. In this study, pulp exposure stimulated tooth inflammation, while the expressions of TNF-α, IL-1β, PGE2, and COX-2 were up regulated. It was consistent with a previous study, since Ozdemir *et al*.^23^ raised that proinflammatory cytokines (IL-1α, IL-1β, IL-6, and TNF-α) suffer higher expressions in pulp with pulpitis than normal. However, PAV treatment reversed it with dose dependence. This study has shown that PAV could inhibit the inflammation of pulpitis mice.

Periodontal ligament stem cells (PDLSCs), a type of mesenchymal stem cells, were studied for periodontal regeneration. The process of osteogenic differentiation of PDLSCs was reported that influenced by inflammatory microenvironments^24^. The results indicated that pulp exposure leads to osteoclast formation and osteogenic differentiation inhibition. However, 40 mg/kg PAV significantly reduced osteoclast number. Furthermore, the expressions of BMP-2, Runx2, osterix, and osteocalcin bear out the TARP results in gene levels. As reported, Runx2, osterix, and osteocalcin are all osteoblast differentiation markers in a mass of studies, and BMP-2 is a strong inducer^25^^,^ 26. It suggests that PAV has the capacities of stabilizing osteoblast differentiation and inhibiting osteoclast formation.

Runx2 is a key transcription factor of osteogenic differentiation, leading to the BMP-2 gene up-regulation^27^. However, Runx2 is directly stimulated by the Wnt/β-catenin signaling pathway, finally leading to the formation of the bone^28^. GSK3β is a negative regulator of the Wnt/β-catenin signaling pathway. It often binds to β-catenin, Axin, and the tumor-suppressive adenomatous polyposis coli (APC) protein in the absence of Wnt, and forms a degradation complex^29^. Meanwhile, GSK3β is an important signaling molecule in Wnt/β-catenin signaling, which plays a critical role in controlling the nucleation of β-catenin^29^. In addition, other studies have confirmed that proinflammatory cytokines, such as TNF-α, could stimulate the activation of the classical Wnt signaling pathway, and negatively regulates the process of osteogenic differentiation in PDLSCs^30^. The Wnt/β-catenin signaling pathway activation is strongly associated with negative feedback in osteogenic differentiation. In this study, Wnt3a protein and the p-β-catenin/β-catenin ratio were significantly down-regulated by pulp exposure and increased by PAV in a dosed manner. On the contrary, the p-GSK3β/GSK3β ratio exhibited a different state from the p-β-catenin/β-catenin ratio. It means that pulp exposure stimulated the activation of the Wnt/β-catenin signaling pathway, but PAV suppresses it.

## Conclusion

PAV showed anti-inflammation and inhibition of osteoclast formation in pulpitis mice. PAV may act as a pulpitis treatment drug, reduces the inflammatory factors released, inhibits the formation of osteoclasts by suppressing the activation of the Wnt/β-catenin signals, and promotes the enhancement of dental bone density.
